# Renal Sarcoidosis With Increased 1,25(OH)2D3 Production via CYP27B1-Expressing Epithelioid Granuloma: A Case Report and Review

**DOI:** 10.7759/cureus.95419

**Published:** 2025-10-26

**Authors:** Shinya Harada, Makoto Abe, Yuko Ono, Kazuyuki Ishida, Akihiro Tojo

**Affiliations:** 1 Department of Nephrology and Hypertension, Dokkyo Medical University, Mibu, JPN; 2 Department of Diagnostic Pathology, Dokkyo Medical University, Mibu, JPN

**Keywords:** 1'25-dihydroxyvitamin d3, acute kidney injury, cyp27b1, epithelioid granuloma, hypercalcemia, multinucleated giant cell, renal sarcoidosis

## Abstract

Sarcoidosis can cause acute kidney injury (AKI) through hypercalcemia and granulomatous interstitial nephritis. However, the detailed mechanisms linking intra-renal granuloma activity and AKI are not fully elucidated. A 69-year-old woman presented with AKI associated with elevated 1,25(OH)_2_D_3_ levels. A renal biopsy revealed tubulointerstitial nephritis with the presence of epithelioid granulomas and multinucleated giant cells. Immunohistochemistry showed that these cells expressed 25-hydroxyvitamin D-1 alpha hydroxylase (CYP27B1). Glucocorticoid therapy rapidly improved 1,25(OH)_2_D_3_ levels at initiation, but these levels fluctuated in relation to renal function during glucocorticoid tapering. Plasma 1,25(OH)_2_D_3 _may be produced by CYP27B1 expressed in renal granulomas and serves as an indicator for the treatment of renal sarcoidosis.

## Introduction

Sarcoidosis is a systemic inflammatory disease of unknown etiology. In 90% of cases, the disease presents with non-caseating granulomas in the lungs and mediastinal lymph nodes; however, it is known to affect any organ in the body [1,2]. In particular, hypercalcemia is observed in 10% of patients with sarcoidosis and has been shown to cause acute kidney injury (AKI) [1,3]. This is thought to be due to an increase in 1,25(OH)_2_D_3_; however, the cause and pathological significance remain unclear [4]. As demonstrated in a previous study that highlighted how microscopic analysis can uncover systemic mechanisms of organ injury across diverse disease contexts [5], histopathological examination plays a pivotal role in elucidating the pathophysiological mechanisms underlying sarcoidosis and its organ-specific manifestations. Detailed tissue evaluation not only confirms granulomatous inflammation but also clarifies associated metabolic or immune-mediated alterations contributing to renal dysfunction. We report a case of renal sarcoidosis in which expression of 25-hydroxyvitamin D-1 alpha hydroxylase (CYP27B1) in epithelioid granulomas and multinucleated giant cells led to 1,25(OH)_2_D_3_ elevation, resulting in AKI.

## Case presentation

A 69-year-old woman visited our hospital complaining of edema in her eyelids and legs. She had been in good health, except for surgeries for cholelithiasis and appendicitis seven years prior. She had normal renal function and no family history of kidney disease. Her blood pressure was 120/70 mmHg, heart rate was 74/min, body temperature was 36.7°C, and oxygen saturation (SpO_2_) was 98% (on room air). There was mild edema in her legs, but heart and lung sounds were normal, and there was no obvious cervical lymph node swelling or goiter. Urinalysis revealed a low specific gravity of 1.009, consistent with hyposthenuria, and no proteinuria or hematuria was observed. Urinary N-acetyl-β-D-glucosaminidase (NAG) was normal at 3.6 IU/L, but urinary β_2_-microglobulin (β_2_-MG) was elevated at 5,350 µg/L. Renal function was declining, with a serum creatinine (Cr) of 1.78 mg/dL and an estimated glomerular filtration rate (eGFR) of 22.6 mL/min/1.73 m^2^, calculated using the Japanese Society of Nephrology (JSN) equation for females (eGFR = 194 × Cr^-1.094 ^× Age^-0.287^ × 0.739). The corrected serum calcium (cCa) level was slightly elevated at 10.7 mg/dL with inorganic phosphorus (IP) of 4.5 mg/dL associated with an elevated 1,25(OH)_2_D_3_ level of 134 pg/mL (normal range: 20-60 pg/mL). While the intact parathyroid hormone (iPTH) level was suppressed at 12.0 pg/mL, the parathyroid hormone-related peptide (PTHrP) was less than 1.0 pmol/L. Elevated levels of plasma lysozyme of 30.0 µg/mL (4.3-11.5 µg/mL), soluble interleukin-2 receptor (sIL-2R) of 3,564 U/mL (220-530 U/ml), and angiotensin-converting enzyme (ACE) of 39.2 IU/L (8.3-21.4 IU/L) were consistent with sarcoidosis. Table 1 presents the laboratory findings for this case.

**Table 1:** Laboratory findings. WBC: white blood cell count; RBC: red blood cell count; Hb: hemoglobin; Plt: platelet count; BUN: blood urea nitrogen; Cr: serum creatinine; eGFR: estimated glomerular filtration rate; Na: sodium; K: potassium; Cl: chloride; cCa: corrected calcium; IP: inorganic phosphate; iPTH: intact parathyroid hormone; PTHrP: parathyroid hormone-related peptide; sIL-2R: soluble interleukin-2 receptor; ACE: angiotensin-converting enzyme; NAG: urinary N-acetyl-β-D-glucosaminidase; β_2_-MG: β_2_-microglobulin.

Investigation	Patient value	Normal range
Complete blood count		
WBC (×10^9^/L)	7.00	3.30-8.60
RBC (×10^12^/L)	3.50	3.86-4.92
Hb (g/dL)	10.8	11.6-14.8
Plt (×10^9^/L)	318	158-348
Serum biochemistry		
Albumin (g/dL)	3.9	4.1-5.1
BUN (mg/dL)	38.1	8-20
Cr (mg/dL)	1.86	0.46-0.79
eGFR (mL/min/1.73m^2^)	22.6	>60
Uric acid (mg/dL)	6.9	2.6-7.0
Na (mmol/L)	137	138-145
K (mmol/L)	4.5	3.6-4.8
Cl (mmol/L)	102	101-108
cCa (mg/dL)	10.7	8.8-10.1
IP (mg/dL)	4.5	2.7-4.6
1,25(OH)_2_D_3_ (pg/mL)	134	20-60
iPTH (pg/mL)	12.0	10-65
PTHrP (pmol/L)	<1.0	<1.3
Lysozyme (µg/mL)	30.0	4.3-11.5
sIL-2R (U/mL)	3,564	220-530
ACE (IU/L)	39.2	8.3-21.4
Urinalysis		
Specific gravity	1.009	1.010-1.030
Urinary protein excretion (g/gCr)	0.10	<0.15
Urinary red blood cells (/HPF)	<1	<5
Urinary white blood cells (/HPF)	1-4	<5
NAG (IU/L)	3.6	<8.0
Urinary β2-MG (µg/L)	5,350	<230

Computed tomography scans of the chest and abdomen revealed significant swelling of the mediastinal and right hilar lymph nodes. A bronchoscopic lymph node biopsy showed non-caseating epithelioid cell granulomas, indicating a diagnosis of pulmonary sarcoidosis.

**Figure 1:**
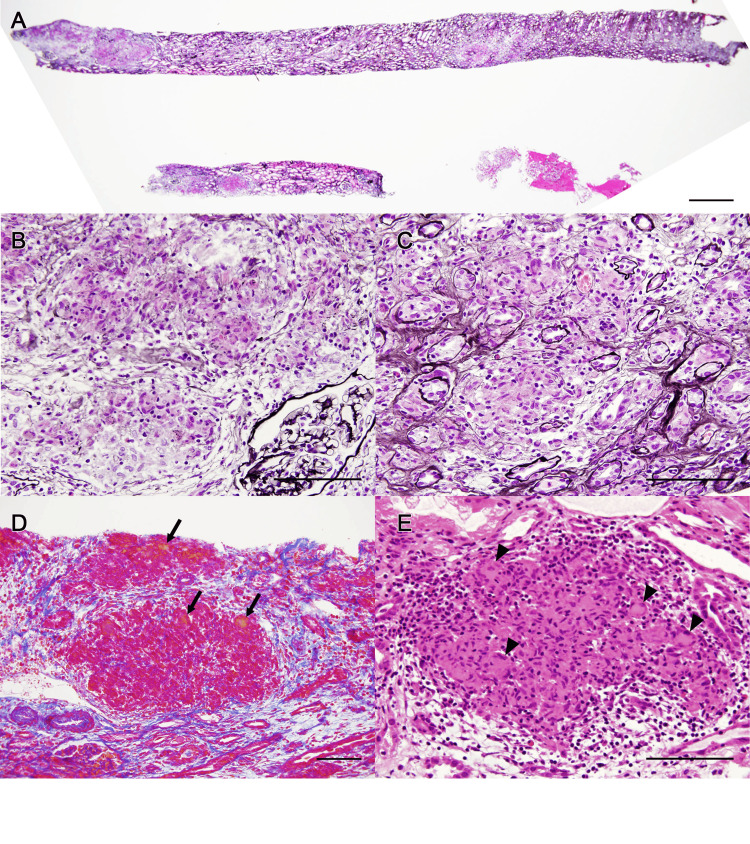
Light microscopy of renal biopsy samples. (A-C) PAM staining shows cellular proliferation in the tubulointerstitium without changes in the glomerulus. (D) AZAN staining shows accumulation of orange substance (arrow). (E) HE staining shows epithelioid cell granulomas and multinucleated giant cells (arrowhead). Bars: A = 1 mm, B-E = 100 µm. PAM: periodic acid methenamine silver; AZAN: azocarmine, aniline blue, and orange G; HE: hematoxylin and eosin.

**Figure 2:**
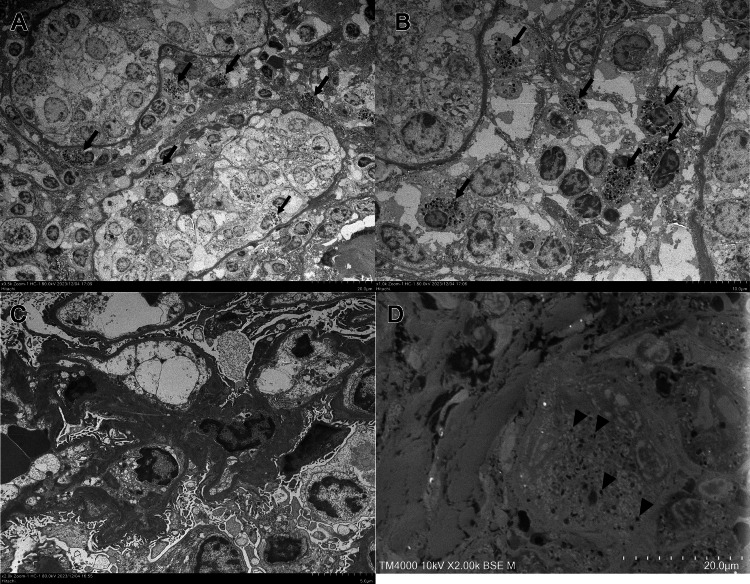
Electron microscopy of renal biopsy samples. (A, B) Transmission electron microscopy shows accumulation of granular material in interstitial infiltrating cells (arrow). (C) No electron-dense deposits in the glomerulus or disappearance of the foot process are observed. (D) Low-vacuum scanning electron microscopy shows similar granular material in multinucleated giant cells (arrowhead).

**Figure 3:**
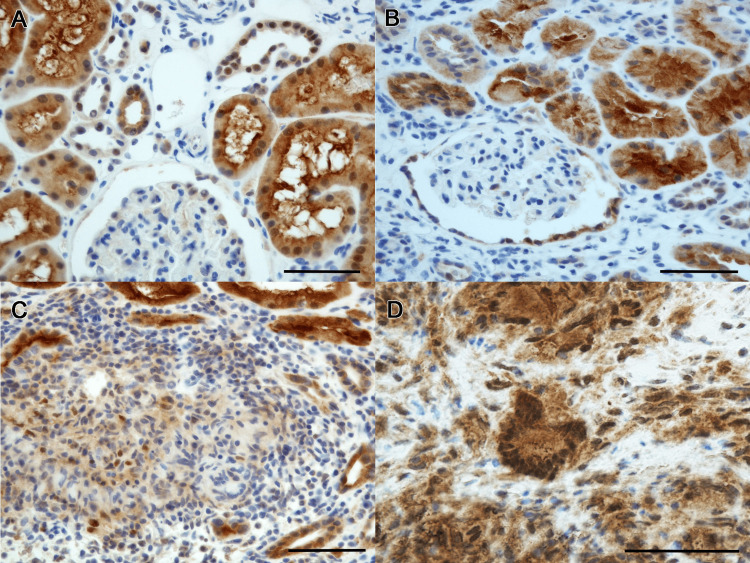
Immunohistochemistry for 25-hydroxyvitamin D-1 alpha hydroxylase (CYP27B1) using anti-human CYP27B1 antibody (Sigma-Aldrich, Merck KGaA, Darmstadt, Germany) with 1:400 dilution. Normal control staining in proximal tubules in thin basement membrane disease (A). In the present case (B-D), strong CYP27B1 expression is observed in epithelioid cell granulomas and multinucleated giant cells (C, D) in addition to proximal tubules (B). Bars: 100 µm.

Because of progressive renal dysfunction, a history of pulmonary sarcoidosis, and elevated tubular injury markers, a renal biopsy was performed. The renal biopsy revealed tubulointerstitial nephritis associated with epithelioid granulomas and multinucleated giant cells. Six glomeruli were normal, with one showing global sclerosis (Figure 1). AZAN (azocarmine, aniline blue, and orange G) staining revealed an accumulation of orange-stained substance in the granulomatous areas (Figure 1D). Immunofluorescence staining was negative for IgG, IgA, IgM, and C3 in the glomeruli and lesions. Electron microscopy revealed no foot process effacement or electron-dense deposits (Figure 2C), and the epithelioid granulomas and multinucleated giant cells in the interstitium contained granular material 5-10 nm in size (Figures 2B, 2D). CYP27B1 renal expression was observed in the proximal tubules in thin basement membrane disease as a control (Figure 3A). In the present case, CYP27B1 expression was observed in both the epithelioid granuloma cells and the multinucleated giant cells (Figures 3B-3D).

**Figure 4:**
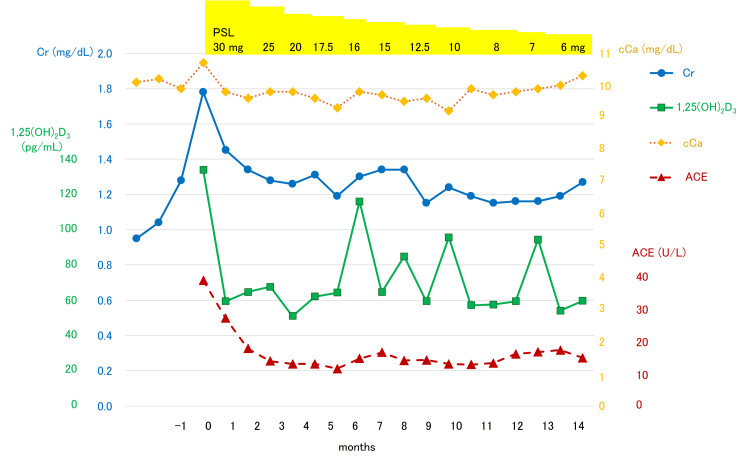
Changes in serum Cr, cCa, 1,25(OH)2D3, and ACE during treatment with prednisolone. PSL: prednisolone; Cr: serum creatinine; cCa: corrected calcium; ACE: angiotensin-converting enzyme.

The patient was diagnosed with granulomatous interstitial nephritis due to sarcoidosis and was treated with 30 mg of prednisolone. The changes in serum Cr, cCa, 1,25(OH)_2_D_3_, and ACE during treatment are summarized in Figure 4. Serum calcium concentrations quickly returned to normal and remained within the normal range, whereas fluctuations in serum 1,25(OH)_2_D_3_ concentrations correlated with fluctuations in serum creatinine concentrations during prednisolone tapering. Mild renal dysfunction remained with Cr at 1.27 mg/dL, but serum cCa and ACE normalized with 6 mg prednisolone.

## Discussion

In this case report, we have demonstrated enhanced expression of CYP27B1 in the granuloma cells and multinucleated giant cells of renal sarcoidosis, which is the most plausible mechanism for the observed increased 1,25(OH)_2_D_3_ production. Approximately 10% of sarcoidosis cases are associated with hypercalcemia via increased CYP27B1 [6]. Inactive form of vitamin D_3_ synthesized in the skin is metabolized in the liver to 25(OH)D_3_, which is then hydroxylated by CYP27B1 to the active form of 1,25(OH)_2_D_3_. It has been believed that increased extrarenal CYP27B1 leads to overproduction of 1,25(OH)_2_D_3_, causing hypercalcemia in sarcoidosis [7]. In the present case, we showed an increased expression of CYP27B1 in renal sarcoidosis.

Renal sarcoidosis is a rare form of sarcoidosis characterized by granulomatous inflammation confined to the renal cortex and accounts for 0.7-0.9% of sarcoidosis cases [8]. The degree of renal dysfunction varies in sarcoidosis treated with glucocorticoid therapy [7]. The causes of kidney damage include not only granulomatous tubulointerstitial nephritis, but also dehydration or renal stones due to hypercalcemia [8,9].

CYP27B1 is primarily expressed in the proximal tubules of the kidney, and in the placenta during pregnancy [10,11]. It is also expressed in granulomatous conditions, in which macrophages activate vitamin D_3_ by producing CYP27B1 outside the kidney [12]. Cases of renal sarcoidosis with increased 1,25(OH)_2_D_3_ and AKI are summarized in Table 2. In some of these cases, the serum calcium concentration was not high enough to cause dehydration [13-17]. Additionally, glucocorticoid treatment reduced the serum calcium to the normal level, but renal function was not restored to the normal level in some cases, including the present case [15-17]. Thus, the persistence of renal dysfunction after the normalization of serum calcium suggests that AKI in renal sarcoidosis cannot be attributed solely to hypercalcemia and is likely also due to direct granulomatous inflammation. We revealed increased CYP27B1 expression in epithelioid granulomas and multinucleated giant cells (Figures 3C, 3D) with secretory granules (Figures 2B, 2D), which may release 1,25(OH)_2_D_3_. Glucocorticoid therapy inhibits granuloma formation and reduces CYP27B1 expression in granulomatous areas of cutaneous sarcoidosis [18]. Therefore, we believe that serum 1,25(OH)_2_D_3_ can indicate granuloma formation and renal sarcoidosis disease activity. Sustained elevation of 1,25(OH)_2_D_3_ requires chronic treatment [19], and renal damage increases when 1,25(OH)_2_D_3_ is 30 pg/mL or higher [20]. In fact, a re-elevation of 1,25(OH)_2_D_3_ was observed during the gradual prednisolone tapering in the present case. Therefore, we propose that serum 1,25(OH)_2_D_3_ levels can be an important indicator for the treatment of renal sarcoidosis.

**Table 2:** Renal sarcoidosis with acute kidney injury and hypercalcemia via increased 1,25(OH)2D3. Cr: creatinine; Ca: calcium; PSL: prednisolone; eGFR: estimated glomerular filtration rate.

Author	Age, sex	Cr (mg/dL)	eGFR (mL/min/1.73m^2^)	Ca (mg/dL)	1,25(OH)_2_D_3_ (pg/mL)	Therapy	Post Cr (mg/dL)	Post eGFR (mL/min/1.73m^2^)	Post Ca (mg/dL)	Post 1,25(0H)_2_D_3_ (pg/mL)
Manjunath et al. (2013) [13]	43, male	2.7	-	11.2	75	PSL 40 mg	1.9	-	-	-
Unsal et al. (2013) [14]	30, male	2.2	-	11.5	34	PSL 1 mg/kg, chloroquine 250 mg	1.3	-	11.3	37
Toriu et al. (2019) [15]	54, female	-	67.4	9.3	68.2	PSL 20-40 mg	-	67.1	9.3	47.5
	46, male	-	44.7	9.1	58.0	PSL 20-40 mg	-	45.6	8.9	39.0
	72, male	-	31.8	10.3	41.1	PSL 20-40 mg	-	32.1	10.0	28.0
	20, male	-	34.2	10.4	79.3	PSL 20-40 mg	-	28.7	9.5	48.3
	82, male	-	4.2	9.6	49.4	PSL 20-40 mg	-	4.3	8.4	23.2
	36, male	-	62.5	10.0	42.1	PSL 20-40 mg	-	52.5	9.4	52.8
	75, female	-	10.2	12.5	34.6	PSL 20-40 mg	-	24.9	9.8	23.7
	76, male	-	5.5	9.7	33.0	PSL 20-40 mg	-	9.0	9.2	18.0
	74, female	-	46.8	9.7	30.0	PSL 20-40 mg	-	56.8	9.4	18.9
Iwazu et al. (2021) [16]	42, male	1.82	-	12.0	112	PSL 35 mg	1.1	-	9.9	60
Mio et al. (2022) [17]	62, male	2.27	24.2	13.2	158	PSL 25 mg	1.5	-	10.1	32.0
Present case	69, female	1.78	22.6	10.7	134	PSL 30 mg	1.27	32.6	10.3	59.6

## Conclusions

In this case, renal dysfunction persisted even after serum calcium levels were normalized. Plasma 1,25(OH)_2_D_3_ concentrations increased during the course of prednisolone tapering. We directly demonstrated CYP27B1 expression within renal granulomas, providing a pathological basis for the elevated 1,25(OH)_2_D_3_. Monitoring 1,25(OH)_2_D_3_ levels may therefore serve as a valuable indicator of granulomatous disease activity to guide treatment in renal sarcoidosis.
